# Can Social History Variables Predict Prison Inmates' Risk for Latent Tuberculosis Infection?

**DOI:** 10.1155/2012/132406

**Published:** 2012-12-23

**Authors:** Tyler E. Weant, Abigail Norris Turner, Maureen Murphy-Weiss, David M. Murray, Shu-Hua Wang

**Affiliations:** ^1^Division of Epidemiology, College of Public Health, The Ohio State University, Columbus, OH 43210, USA; ^2^Division of Infectious Diseases, Department of Internal Medicine, The Ohio State University, Columbus, OH 43210, USA; ^3^Tuberculosis Program, Ohio Department of Health, Columbus, OH 43210, USA; ^4^Biostatistics and Bioinformatics Branch, Division of Epidemiology, Statistics, and Prevention Research, The Eunice Kennedy Shriver National Institute of Child Health and Human Development, National Institutes of Health, Rockville, MD 20892, USA

## Abstract

Improved screening and treatment of latent tuberculosis infection (LTBI) in correctional facilities may improve TB control. The Ohio Department of Rehabilitation and Correction (ODRC) consists of 32 prisons. Inmates are screened upon entry to ODRC and yearly thereafter. The objective of the study was to determine if social history factors such as tobacco, alcohol, and drug use are significant predictors of LTBI and treatment outcomes. We reviewed the medical charts of inmates and randomly selected age-matched controls at one ODRC facility for 2009. We used a conditional logistic regression to assess associations between selected social history variables and LTBI diagnosis. Eighty-nine inmates with a history of LTBI and 88 controls were identified. No social history variable was a significant predictor of LTBI. Medical comorbidities such as asthma, rheumatoid arthritis, and hepatitis C were significantly higher in inmates with LTBI. 84% of inmates diagnosed with LTBI had either completed or were on treatment. Annual TB screening may not be cost-effective in all inmate populations. Identification of factors to help target screening populations at risk for TB is critical. Social history variables did not predict LTBI in our inmate population. Additional studies are needed to identify inmates for the targeted TB testing.

## 1. Introduction

One-third of the world's population is infected with *Mycobacterium tuberculosis* [1]. While most of these infections occur in resource-poor regions, tuberculosis (TB) has been observed among prison inmates in the United States (US) [2]. Incarceration itself is a risk factor for incident TB [3]. In 2009, 4.2% of TB cases in the US occurred in individuals who were residents of a correctional facility at the time of diagnosis [4]. The transient nature of the prison population can adversely impact the public health of the general population [5]. If TB is not properly detected and treated during incarceration, inmates may develop active TB disease and subsequently transmit TB to fellow inmates, staff, or members of the community to which they return upon release. The CDC released guidelines in 2006 for TB screening, prevention, and treatment in correctional facilities [6].

In 2001, the Ohio Department of Rehabilitation and Correction (ODRC) reported 5 cases of active TB disease among its inmates. These five cases were linked to one another epidemiologically and by molecular genotyping [7]. The incidence rate of active TB disease for the prison inmate population in Ohio in 2001 was 11.4 cases per 100,000, compared to 2.0 per 100,000 in the general Ohio population during the same year. In an effort to improve TB screening and prevent another outbreak, in 2004 ODRC implemented statewide standardized TB screening for all 32 ODRC sites on the same day. This new screening is in addition to the initial screening new inmates receive upon entrance to an ODRC facility. All inmate movements into and out of the facilities are suspended during the screening period. In 2009, 0.06% (28/46,875) of inmates screened had a positive tuberculin skin test (TST) (Table 1). A more cost-effective method may be needed for annual TB screening in prison populations with low prevalence for LTBI.

The goals of the current study were to review the statewide annual TB screening protocol implemented by ODRC and to perform a detailed examination of the TB screening protocol at one ODRC facility. We also sought to determine if social history factors such as history of tobacco, alcohol, and intravenous (IV) and non-IV drug use are significantly associated with LTBI status, to see whether these could be used to predict which inmates were more likely to be diagnosed with LTBI. Finally, we sought to evaluate LTBI treatment outcomes in the correctional facility. 

## 2. Methods

We reviewed ODRC's annual TB screening protocol for TSTs and results for 2004–2009. We reviewed the medical charts of inmates with LTBI and randomly selected age-matched controls at Pickaway Correctional Institute (PCI), one of the 32 ODRC facilities for 2009. The PCI is a moderate security, male-only prison which housed approximately 2,400 inmates during the study period. We used a conditional logistic regression to assess associations between selected social history variables and LTBI diagnosis.

Medical charts were reviewed and data on past medical and social history, laboratory and radiology results, information on LTBI treatment (medication, dosage, side effects, and completion status), and clinical data from inmates' baseline medical exam conducted at the initiation of incarceration, annual TB screening exams, and TB clinic notes were obtained. Social history variables of tobacco, alcohol, and drug use were collected from a self-reported mental health examination questionnaire. Substance use was coded dichotomously as ever used or never used for the analysis. For LTBI treatment, the ODRC protocol recommended a regimen of 900 mg of isoniazid (INH) administered twice weekly for 9 months for LTBI. An alternative treatment of daily rifampin 600 mg for 4 months was recommended to inmates who were intolerant of INH. Treatments were administered by a directly observed therapy (DOT). Documentation of TB treatment during or prior to incarceration was required for treatment to be considered complete.

A reference population of 88 age-matched control inmates with negative TSTs was randomly chosen from the inmate population at the correctional facility four months after data collection for cases had been completed. Information on medical and social history for this population was collected using the same methods described above. Because of the inability to find an age-matched control for one of the 89 LTBI cases, one LTBI case was not included in the conditional logistic regression analyses. 

Ethical approval for the study was obtained from the Institutional Review Boards at both The Ohio State University and ODRC. 

### 2.1. Analysis

All statistical analyses were conducted using STATA (Version 10) or SAS (Version 9.2). Fisher's exact test was utilized for medical history comparisons between the cases and controls. We used a conditional logistic regression to assess unadjusted associations between various social history variables and odds of LTBI. The threshold for statistical significance was *α* = 0.05.

## 3. Results

Between 2004 and 2009, ODRC performed TST screening tests on all inmates with history of negative TST at entry. Positive TSTs during the annual screening decreased from 0.41% in 2004 to 0.06% in 2009 (Table 1). During the six years assessed, a total of 603 (0.23%) of 258,913 inmates tested had positive TST results or converted to positive after their initial entry screening (Table 1). Similar overall results were seen at PCI where 27 (0.24%) of 11,133 TSTs conducted were positive during this time period.

**Figure 1:**
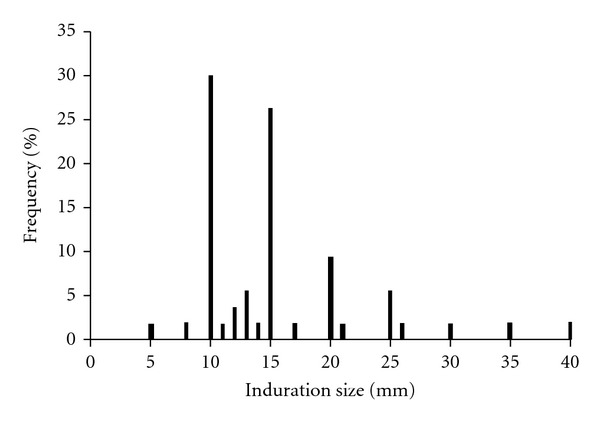
Distribution of TST induration size for inmates diagnosed with LTBI^a,b,c^. ^a^Two inmates with TSTs <10 mm had previously tested positive (>10 mm) and had been diagnosed with LTBI. ^b^One TST result listed as 17–20 mm was input at 17 mm. ^c^One TST result listed as >20 mm was input at 21 mm. LTBI: latent tuberculosis infection; TST: tuberculin skin test.

At PCI, 89 inmates had a positive TST result recorded in their medical chart for 2009. Of the 89 inmates with positive TST, 37 (42%) tested positive for the first time upon entrance to the facility for their current incarceration and 52 inmates had a positive TST prior to their current incarceration. Distribution of TST induration sizes is shown in Figure 1. No inmates tested positive during the annual skin test. These individuals ranged in age from 23 to 66 years with a mean age of 40 years (Table 2). Nine percent of inmates with a positive TST who were examined experienced one or more symptoms consistent with active TB disease such as fever, fatigue, night sweats, and/or unintentional weight loss (Table 2). However, medical evaluation and chest radiographs showed no evidence of active TB disease and these individuals were diagnosed with LTBI. 

### 3.1. Social History Variables and LTBI

Use of tobacco, alcohol, and drug were not significantly associated with LTBI in this age-matched prison population (Table 3). Although we observed somewhat increased odds of LTBI for tobacco use (OR = 1.08, 95% CI, 0.49–2.37), alcohol use (OR = 1.44, 95% CI, 0.62–3.38), and IV drug use (OR = 1.44, 95% CI, 0.62–3.38), these associations were not statistically significant. In contrast, we observed *decreased, *nonstatistically significant odds of LTBI for non-IV drug users (OR = 0.50, 95% CI, 0.23–1.07, *P* = 0.07).

### 3.2. Medical History and LTBI

Inmates with LTBI had significantly higher rates of asthma (21% versus 8%, *P* = 0.03) and rheumatoid arthritis (11% versus 1%, *P* < 0.01) than the control population (Table 4). Cases also had somewhat higher rates of hepatitis C (20% versus 10%, *P* = 0.09). We observed no significant differences between LTBI cases and controls in the prevalence of diabetes, nonrheumatoid arthritis, hepatitis B, cancer, hypertension, sexually transmitted diseases (including HIV), or mental health disorders.

### 3.3. LTBI Treatment

In total, 75 of the 89 inmates (84%) who were diagnosed with LTBI had completed treatment or were currently receiving treatment (Table 5). Among the 37 individuals diagnosed upon entrance to the prison facility, 27 (73%) were started on treatment and either completed or were still on treatment at the time of data collection. Among the 27 men treated at the prison, three experienced adverse reactions (chills, headaches, and nausea) during treatment with INH. Treatment was interrupted for two of these cases: one completed and the other refused further treatment. An adverse reaction was documented for one additional case treated prior to incarceration and the inmate completed an alternative regimen of rifampin in prison. HIV test results were documented for 83 of the 89 (93%) LTBI cases. Two were found to be HIV positive; both were receiving highly active antiretroviral therapy.

## 4. Discussion

TB outbreaks among prison inmates have been reported and TB screening is a major component of TB control programs [2, 4, 8, 9]. A correctional facility has “nonminimal” risk for TB if a case of active infectious TB disease occurred in the facility in the last year, the inmates in the facility have TB risk factors such as HIV or are new immigrants (in the US 5 or fewer years) from a TB endemic country, or the employees are at risk for TB [6]. Symptomatic assessment of active TB disease and LTBI screening with TST or interferon gamma release assays (IGRA) are recommended at prison entry. The CDC guidelines also recommend annual screening for long-term inmates and all employees who have a negative TST or IGRA result. If the conversion rate increases or a TB outbreak is identified, increased TB testing is needed [6].

In reviewing the annual TST screening results across all ODRC facilities, the percentage of positive TST results decreased yearly. During the six years of testing, the TST conversion rate was 0.23% (Table 1). ODRC appears to have a lower TST conversion rate than other prison populations in the US [10, 11].

Due to geographic constraints, the researchers were only able to go to one of the 32 ODRC facilities to perform chart review on inmates with positive TST conversions and no electronic medical records were available for reviews off site. Similar to ODRC's overall annual TST conversion rate, the conversion rate at PCI also decreased from 2004 to 2009 (Table 1). However, unlike ODRC's continuous decline, PCI experienced fluctuations: an increase to 0.69% was seen in 2007. The cause of the increase is unknown. No outbreaks were reported at this time. Interestingly, the medical chart review of the 89 LTBI cases identified at PCI in 2009 showed that there were no conversions for the year; 42% tested positive at entry and 58% had a history of being positive prior to entry. 

The goal of TB screening is to detect active TB disease; these findings suggest that TB screening at facility entry may identify a majority of LTBI cases and should continue for both minimal and nonminimal TB risk facilities. However, the low rate of TST conversion also suggests that yearly screens in the entire inmate population may not be warranted. An improved algorithm for identifying high-risk individuals for periodic screening, particularly for minimal risk facilities, should be evaluated. 

The previous literature suggests that inmates with a history of alcohol, tobacco, or drug use may be at increased risk for exposure to TB and thus at risk for LTBI [6]. A history of alcohol use may increase the likelihood of TB transmission in impoverished populations that drink in social groups [12]. Increased alcohol intake can impair immune function, resulting in increased susceptibility to TB after exposure [13]. Similarly, tobacco use, specifically smoking, can damage lung tissue and lead to greater TB risk. A positive association between tobacco use and LTBI has previously been identified in a meta-analysis [14]. While past studies found a significant increase in LTBI among those who had a ever smoked, the included study populations had substantially lower prevalence of smoking compared to the inmate population in the present investigation. 

In contrast to past studies, we did not observe a significant association between IV drug use and LTBI. In a recent review of drug use and TB, mixed results were found when investigating IV drug use as a risk factor for positive TST [15]. Some studies detected a strong association between IV drug use and LTBI compared to a non-IV drug using populations, without comparing IV drug users to nondrug users [16–18]. In our study, we observed nonsignificantly increased odds of LTBI for tobacco, alcohol, and intravenous drug users. This result may be due to the high prevalence of substance abuse, specifically tobacco and alcohol use, among prison inmates. Similar to an earlier study in crack smokers, we found a borderline significant inverse relationship between non-IV drug use and LTBI in our study population [17]. 

Interestingly, we did observe significant differences in medical comorbidities between inmates with LTBI and the non-LTBI control population. Inmates with LTBI had a significantly higher prevalence of asthma and rheumatoid arthritis. A previous study suggested that *M. tuberculosis* infection during childhood could modify immunogenic responses which then reduced incidence of atopic disorders such as asthma [19]. It is unknown whether inmates included in the study were diagnosed with asthma as children or adults. It is well documented that individuals with COPD are at increased risk for TB disease [20].

The prevalence of hepatitis C in the LTBI population was marginally higher than in the control group. This finding is consistent with a previous study of US Veterans Affairs hospitals which identified a significantly higher prevalence of TB among hepatitis C infected patients [21]. Because a higher prevalence of arthritis has previously been identified with hepatitis C infection, increased prevalence of rheumatoid arthritis and presentation of rheumatoid-like symptoms among those with hepatitis C in this study are not unexpected [22, 23]. It is known that patients receiving immunosuppressive agents such as prednisone or TNF-*α* blockers are at increased susceptibility to TB upon exposure; these individuals also have a higher risk of reactivation of *M. tuberculosis* once infected [24]. This may account for increased diagnosis of rheumatoid arthritis in inmates with LTBI; however, due to the small sample size, further evaluations are needed.

Among inmates diagnosed with LTBI, 84% had completed or were currently receiving treatment. Although adverse treatment reactions occurred at approximately twice the rate found in an earlier international investigation, completion rates were higher among inmates than the general public [25, 26]. DOT may offer increased opportunity to report side effects or incarcerated individuals may have a greater motivation to report medication side effects (e.g. a desire to be removed from work or the general prison population). DOT may also lead to the higher completion rate in prison when compared to the general public on self-therapy.

Analysis of TST readings identified a potential terminal digit bias (Figure 1). Approximately 80% of TST results exhibited terminal digits of “5” or “0” and 30% of recorded TST measurements were 10 mm. This phenomenon has been described before and was determined not to be correlated with reduced accuracy in predicting future likelihood of disease [27]. However, the large percentage of individuals with test results of 10 mm, the cutoff for a positive result, could be a concern. Rounding up results of 8 or 9 mm to 10 mm would lead to false positives among the inmate population, leading to unnecessary treatment, potential side effects, and increased cost. Concerns about possible misclassification of LTBI through mismeasured TST induration size could be reduced through the use of IGRAs. IGRAs are not prone to the same subjective interpretation which can bias TSTs [28]. The current CDC guidelines recommend use of either TST or IGRA for general screening [29]. All inmates should have an HIV test performed regardless of the TB screening status. All HIV-infected inmates should receive an annual TB screening test.

Our analysis is limited by the small sample size and retrospective study design. We were able to estimate associations but not causality between hypothesized risk factors and odds of LTBI. Our results may not be generalizable to other ODRC facilities or to other prisons in the US. A larger, multisite prospective study will provide a more comprehensive evaluation of risk factors associated with LTBI, in order to improve the targeted screening of high-risk individuals. The high rate of treatment completion and low rate of side effects are encouraging for continued TB control in correctional facilities. The prison setting may be ideal for the use of the new LTBI short-course regimen of 12 weekly doses of rifapentine 900 mg and INH 900 mg by DOT [30]. This regimen has been shown to be non-inferior to 9 months of INH, but with less hepatotoxicity. 

## 5. Conclusion

Overall, TB screening and treatment practices in Ohio's correctional facility demonstrate the enormous potential of prisons to treat a highly vulnerable population for an infection that might otherwise go unnoticed, ultimately leading to increased morbidity and mortality in the nation. Although TB screening at prison entry is needed, continued annual screening may not be warranted in populations at low risk for TB and may not be cost-effective. Ideally, TB risk factors could be identified and used for the targeted annual screening in correctional facilities. Social history variables such as history of tobacco, alcohol, or drug use were not significantly associated with an increased diagnosis of LTBI in our population, but certain medical comorbidities were significantly more prevalent in prisoners with LTBI than in control prisoners. Additional studies are needed to identify which inmates should be targeted for annual TB testing.

## Figures and Tables

**Table 1:** Annual TB surveillance testing of incarcerated individuals, Ohio, 2004–2009^a^.

Year	ODRC			PCI		
	Tested	Positive TST		Tested	Positive TST	
		*N*	(%)		*N*	(%)
2004	39,090	159	(0.41)	1,398	8	(0.57)
2005	39,448	132	(0.33)	1,362	0	(0)
2006	42,452	117	(0.28)	2,257	3	(0.13)
2007	44,812	91	(0.20)	2,171	15	(0.69)
2008	46,236	76	(0.16)	2,207	1	(0.05)
2009	46,875	28	(0.06)	1,738	0	(0)

Total	258,913	603	(0.23)	11,133	27	(0.24)

^a^Inmates with previously positive TST were not retested.

ODRC: Ohio Department of Rehabilitation and Correction; PCI: Pickaway Correctional Institution; TST: tuberculin skin test.

**Table 2:** Medical examination of 89 inmates with positive TSTs.

Cases (*N*)	89	
Age		
Mean (median)	40 (39)	
Range	23–66	
Symptoms	*N*	(%)
Fever		
Yes	1	(1)
No	61	(69)
Missing	27	(30)
Cough		
Yes	0	(0)
No	62	(70)
Missing	27	(30)
Unintentional weight loss		
Yes	3	(3)
No	63	(71)
Missing	23	(26)
Fatigue		
Yes	2	(2)
No	61	(69)
Missing	26	(29)
Night sweats		
Yes	2	(2)
No	60	(68)
Missing	27	(30)
Chills		
Yes	0	(0)
No	62	(70)
Missing	27	(30)
Hemoptysis	0	(0)
Yes	0	(0)
No	62	(70)
Missing	27	(30)
Any	6	(9)
Yes	6	(7)
No	60	(68)
Missing	22	(25)

TST: tuberculin skin test.

**Table 3:** Unadjusted associations between social history variables and LTBI in an Ohio correctional facility, 2009.

Variable	Odds ratio (95% CI)	*P* value
Tobacco	1.08 (0.49–2.37)	0.84
Alcohol	1.44 (0.62–3.38)	0.40
Intravenous drug abuse	1.44 (0.62–3.38)	0.39
Nonintravenous drug abuse	0.50 (0.23–1.07)	0.07

LTBI: latent TB infection.

**Table 4:** Baseline medical history of LTBI cases and controls.

Disease	Cases (*n* = 89)		Controls (*n* = 88)		*P* value
	*N*	(%)	*N*	(%)	
Asthma	18	(21)	7	(8)	0.03
Cancer	0	(0)	3	(3)	0.25
Diabetes	4	(5)	2	(2)	0.68
Hepatitis B	3	(3)	0	(0)	0.25
Hepatitis C	18	(20)	9	(10)	0.09
Arthritis	16	(18)	13	(15)	0.69
Rheumatoid arthritis	10	(11)	1	(1)	<0.01
Hypertension	17	(19)	14	(16)	0.69
Sexually transmitted diseases	9	(10)	12	(14)	0.49
Mental health disorder	6	(7)	12	(14)	0.14
HIV	2	(3)	1	(1)	0.60
Any	51	(57)	46	(52)	0.55

LTBI: latent TB infection.

**Table 5:** Treatment outcomes for inmates at PCI diagnosed with LTBI (*n* = 89).

	LTBI diagnosed upon entrance to current incarceration (*n* = 37)		LTBI diagnosed prior to current incarceration (*n* = 52)		Total (*n* = 89)	
	*N*	(%)	*N*	(%)	*N*	(%)
Currently under care or completed treatment	27	(73)	48	(92)	75	(84)
Experienced adverse reaction	3	(8)	1	(2)	4	(5)
Treatment interrupted	2	(5)	1	(2)	3	(3)
HIV tested	36	(97)	47	(90)	83	(93)
HIV positive^a,b^	0	(0)	2	(4)	2	(2)

^a^The number of inmates tested for HIV was used as the denominator for percentages.

^b^Both HIV positive inmates received treatment for HIV.

LTBI: latent TB infection; PCI: Pickaway Correctional Institute.
